# The Role of Low-Dose Oral Methotrexate in Increasing Anti-TNF Drug Levels and Reducing Immunogenicity in IBD

**DOI:** 10.3390/jcm12134382

**Published:** 2023-06-29

**Authors:** Kathryn Demase, Cassandra K. Monitto, Robert D. Little, Miles P. Sparrow

**Affiliations:** 1 Department of Gastroenterology, Alfred Health and Monash University, Melbourne 3004, Australia; 2 Department of Pharmacy, Alfred Health, Melbourne 3004, Australia

**Keywords:** methotrexate, oral, infliximab, adalimumab, concomitant immunomodulator, pharmacokinetics, inflammatory bowel disease

## Abstract

Concomitant immunomodulation is utilised in combination with anti-TNF therapy for IBD primarily to increase drug levels and prevent anti-drug antibody formation. Whilst thiopurines have traditionally been the immunomodulator of choice in IBD populations, there are concerns regarding the long-term safety of the prolonged use of these agents: particularly an association with lymphoproliferative disorders. Given this, we have explored the existing literature on the use of low-dose oral methotrexate as an alternative immunomodulator for this indication. Although there is a lack of data directly comparing the efficacies of methotrexate and thiopurines as concomitant immunomodulators, the available literature supports the use of methotrexate in improving the pharmacokinetics of anti-TNF agents. Furthermore, low-dose oral methotrexate regimens appear to have comparable efficacies to higher-dose parenteral administration and are better tolerated. We suggest that clinicians should consider the use of low-dose oral methotrexate as an alternative to thiopurines when the primary purpose of concomitant immunomodulation is to improve anti-TNF pharmacokinetics.

## 1. Introduction

The role of anti-tumour necrosis factor (anti-TNF) agents within the treatment armamentarium of inflammatory bowel disease (IBD) is well established. Agents such as infliximab (IFX) and adalimumab (ADL) have changed the landscape of medical therapy for both the induction and maintenance of moderate-to-severe ulcerative colitis (UC) and Crohn’s disease (CD) [1,2,3,4,5,6] and now have over 20 years of efficacy and safety data for IBD [7]. Therapeutic drug monitoring (TDM) has become routine in optimising secondary loss of response to anti-TNF therapy in IBD. In particular, TDM of IFX has been shown to improve clinical outcomes and be more cost-effective than empirical dose escalation [8,9]. The data supporting TDM of ADL are, however, less robust [10]. A range of target trough drug levels have been associated with varying depths of clinical, biochemical, and endoscopic remission, as well as perianal fistula healing [11,12,13,14,15,16].

Combination therapy with immunomodulators such as thiopurines (azathioprine (AZA), mercaptopurine (6-MP)) or methotrexate (MTX) increases anti-TNF drug levels and decreases the formation of anti-drug antibodies (ADAs) [17,18,19]. The evidence for the benefits of concomitant immunomodulation with ADL is less consistent than that with IFX [20,21,22,23,24,25]. Thiopurines have traditionally been used as first-line immunomodulators in IBD. Whilst they are effective therapeutic agents both in combination and as monotherapy, their long-term use is associated with serious adverse events (AEs), such as infections, non-melanomatous skin cancers (NMSCs), and lymphoma [26], including hepatosplenic T-cell lymphoma [27]. Although rare, hepatosplenic T-cell lymphoma has high mortality, with a preponderance in young males. Conversely, MTX may have a more tolerable serious side effect profile. It is commonly used in rheumatological conditions both as monotherapy and in combination with anti-TNF agents; however, it is typically reserved for those who are intolerant to thiopurines in IBD [28]. Evidence for its use in IBD is limited to studies of clinical outcomes of parenteral MTX given at varying doses, with few studies addressing the outcomes of using oral MTX to improve anti-TNF pharmacokinetics [29].

This comprehensive literature review examines the current evidence available on the efficacy, safety, and optimal dosing of oral MTX when used as an immunomodulator in combination with anti-TNF therapy for IBD to optimise anti-TNF drug levels and reduce immunogenicity. For when concomitant immunomodulation is used for this purpose, rather than as a second therapeutic agent to treat disease activity, we propose the consideration of low-dose oral (≤12.5 mg/week) MTX, given its favourable safety profile and comparable efficacy.

We conducted a literature search using the PubMed Online database. The search was performed using the following linked search terms: “methotrexate” AND (“anti-TNF” OR “infliximab” OR “adalimumab” OR “golimumab” OR “certolizumab”) AND (“inflammatory bowel disease” OR “Crohn’s disease” OR “ulcerative colitis”) AND (“rheumatoid arthritis” OR “psoriasis” OR “ankylosing spondylitis”) AND (“trough level” OR “drug concentration” OR “anti-drug antibody”). The results were restricted to the English language and original research, presenting data on the efficacy of oral low-dose MTX as a concomitant immunomodulator with anti-TNF therapy, published before 1 May 2023. In total, 68 articles were identified, and their titles and abstracts were screened by one reviewer (KD) to ensure their relevance. After screening, seventeen articles were assessed for eligibility, with an additional four articles added from a review of the reference lists of the selected articles. Studies that investigated the general efficacy of concomitant immunomodulation with thiopurines and MTX but failed to stratify their data by type of immunomodulator were excluded. After review, 10 articles were chosen for discussion (Table 1).

## 2. Pharmacokinetics of Anti-TNFs and the Role of Methotrexate in Increasing Drug Levels and Reducing Immunogenicity

Whilst anti-TNF agents are an effective therapy for IBD, 23–46% of patients treated with standard dosing regimens of IFX or ADL develop secondary loss of response after 12 months [38]. There are multiple proposed pharmacokinetic and pharmacodynamic mechanisms that lead to low drug levels and loss of response. Firstly, clearance of these drugs is increased in active disease. Intestinal inflammation leads to faecal loss of IFX, with higher faecal IFX concentrations found in those with more severe disease and low serum albumin levels [39]. The inverse relationship between baseline albumin levels and anti-TNF clearance [40] may be explained by the interactions between IgG antibodies, such as IFX, or proteins, such as albumin, and the neonatal Fc Receptor (FcRn) [41]. FcRn is found on endothelial cells and plays a role in the recycling and transcytosis of IgG antibodies and serum proteins, preventing them from catabolism and prolonging their half-life. Additionally, elevated C-reactive protein (CRP) levels have been linked to lower IFX trough levels and loss of response in IBD patients [42,43]. The association between these acute phase reactants and reduced drug levels supports the notion that increased anti-TNF clearance correlates with the severity of the disease. 

The most investigated mechanism, however, is the immunogenicity of these agents, which elicit ADA formation against the F(ab)2 fragment of the anti-TNF IgG molecule [44]. The presence of ADAs against IFX has been demonstrated to increase drug clearance [40,45,46]. Whilst all biological drugs induce immunogenicity, they do so at varying degrees. This is partly explained by the structural differences amongst anti-TNF agents, whereby lower immunogenicity rates are associated with the degree of humanisation of molecules [18]. A systematic review and meta-analysis by Thomas et al. found a significant difference in incidences of ADAs against IFX compared to ADL. IFX is a chimeric monoclonal antibody (mAb) comprising murine variable and human Fc regions, whilst ADL is a fully humanised mAb. As expected, the incidence of ADAs against ADL was lower than that with IFX (14.1% vs. 25.3%, respectively; *p* = 0.03) [18]. This partly explains the larger body of evidence supporting the use of immunomodulators in combination with IFX compared to other anti-TNF agents.

### 2.1. Efficacy of Concomitant Immunomodulation in Improving Anti-TNF Levels

Immunomodulators increase the serum concentrations of anti-TNFs. Although the exact mechanism is not well-established, it is presumed that they exert this function by reducing the formation of ADAs. In the SONIC trial, patients with active CD and who received a combination of IFX and AZA had higher IFX levels than those who received IFX monotherapy (3.5 µg/mL vs. 1.6 µg/mL, respectively; *p* < 0.001). These findings were associated with higher corticosteroid-free remission rates in the combination therapy group [47]. Although the advantage of the combination treatment may, in part, be due to an additive immunosuppressive effect of AZA on the underlying disease process, there was also a clear reduction in ADA formation in patients on combination therapy in comparison to monotherapy (0.9% vs. 14.6%, respectively). Post hoc analysis found increasing rates of remission with increasing serum IFX concentrations but no difference between those on combination therapy and those on monotherapy when stratified by drug level [48]. Combination IFX and AZA patients comprised 73.1% of those who achieved the highest quartile of IFX concentrations and only 23.5% of those in the lowest quartile. Furthermore, the addition of immunomodulators can impact outcomes at as early as 4 weeks, which is faster than the onset of their therapeutic efficacy [49]. The benefit of combination therapy has been seen with real-world data from the prospective PANTS UK cohort, which demonstrated that concomitant immunomodulator therapy with thiopurines or MTX prevented ADA formation against IFX and ADL, improved drug levels, and was associated with a higher 54-week clinical remission rate [23].

### 2.2. Mechanism of Action of Methotrexate in Improving Anti-TNF Pharmacokinetics

MTX is a potent folic acid antagonist with proven efficacy in CD treatment due to its anti-inflammatory and pro-apoptotic properties [50]. However, trials in adult patients with UC found no superiority to the placebo in induction or maintenance of remission [51,52]. MTX exerts its cytotoxic effect by blocking dihydrofolate reductase, interfering with DNA synthesis, and inhibiting de novo purine synthesis. These anti-inflammatory pathways may also enhance the efficacy of biologic agents by reducing TNF and IL-12/23 levels [53], even in the absence of any effect in reducing ADAs. The specific effects on immunological processes that may lead to reductions in ADA formation are complex and not fully understood. A distinct immunomodulatory pathway has been observed in preclinical animal models of immunogenicity and may account for MTX’s effects on ADA production. MTX exposure in mice appears to induce T and B cell anergy, thereby blunting their response to antigen stimulation [54,55]. The animals in these studies showed reduced ADA production towards recombinant human proteins when treated with MTX. Furthermore, this response persisted 32 weeks after MTX cessation. Unrelated recombinant human proteins were administered after MTX cessation, and the ADA response was preserved, suggesting that this mechanism is distinct from generalised immunosuppression. Additionally, other immunosuppressive medications, including rapamycin and cyclophosphamide, exhibited no significant effect on the ADA responses, further supporting a unique role of MTX beyond its established immunosuppressive and cytotoxic effects [54]. These “anergic effects” of MTX on T and B cells may explain the mechanism for reducing ADAs that target anti-TNF agents. 

## 3. Efficacy of Methotrexate Compared to Thiopurines as Concomitant Immunomodulators

In contrast to rheumatological conditions, the use of MTX in combination with anti-TNF agents for concomitant immunomodulation in IBD is less common [17]. This may be due to the more limited role MTX plays as a therapeutic agent in adult IBD [56]. Thiopurines, on the other hand, have a robust evidence base for both CD and UC and, as such, are commonly used as monotherapy maintenance agents in many jurisdictions globally. Therefore, when the decision is made to add an anti-TNF agent in patients failing thiopurine monotherapy, most of the patients continue to receive thiopurines for concomitant immunomodulation, with MTX typically reserved for those who either have failed or are intolerant to thiopurines [57]. A preference for thiopurines over MTX is evident across studies that have evaluated the effects of concomitant immunomodulation, with the majority of cohorts showing thiopurine usage rates of 50–70% (Table 1). There may be a trend towards increasing use of MTX as a first-line immunomodulator in the paediatric population due to safety concerns regarding prolonged thiopurine exposure, particularly hepatosplenic T-cell lymphoma. A multi-centre retrospective cohort study found that the proportion of patients who received MTX as their first immunomodulator rose from 14% in 2002 to 60% in 2010 (*p* = 0.005) [57].

### 3.1. Efficacy of Methotrexate for Concomitant Immunomodulation with Anti-TNFs

A review of the literature pertaining to the efficacy of MTX in regard to anti-TNF pharmacokinetics in both rheumatology and IBD has been summarised in Table 1 and Table 2. Overall, MTX has consistently been shown to reduce the formation of ADAs and lead to higher anti-TNF levels. There may be a reduced effect when it is used in combination with ADL compared to with IFX; however, the data on this are mixed [35,36,37]. Two large, multi-centre randomised control trials that investigated the use of MTX in combination with ADL in rheumatoid arthritis and axial spondylarthritis found it to be effective in reducing rates of ADA formation, increasing trough levels, and achieving clinical responses [35,36]. Conversely, a small, randomised control trial in patients with psoriasis found that there were no differences in the ADL levels in those on monotherapy compared to those receiving concomitant MTX [37]. Despite this, the MTX group did have significantly lower rates of ADA formation and achieved more rapid clinical responses than those on ADL monotherapy. Furthermore, a retrospective observational study of 278 CD patients on IFX or ADL with concomitant immunomodulation with either thiopurines (71%) or MTX (29%) found that those who received thiopurines had higher ADL trough levels compared to those who received MTX [32]. Patients on ADL also had higher rates of endoscopic remission when treated in combination with a thiopurine compared to MTX. These differences were not observed in patients on IFX. Further studies comparing the differential effects of MTX and thiopurines when used in combination with ADL would help clarify these conflicting results.

The data on the effects of concomitant MTX on clinical outcomes in IBD are similarly conflicted. Two randomised control trials found no difference in the rate of treatment failure in those on IFX monotherapy compared to combination therapy with MTX; however, one did show an improvement when used in combination with ADL [58,59]. Conversely, a large prospective cohort study found that the combination of IFX and MTX had higher rates of corticosteroid-free deep remission and was less likely to develop secondary non-response compared to IFX monotherapy [19]. The retrospective data on the effects of varying doses of concomitant MTX on clinical outcomes are also mixed but overall suggest that there is no difference between high- and low-dose regimes [30,32,33].

The efficacy of adding a concomitant immunomodulator (thiopurines or MTX) in eliminating ADAs, improving drug levels, and recapturing clinical responses to anti-TNF therapy is more established. Three retrospective studies have investigated the effects of commencing immunomodulators in patients who had developed immunogenic loss of response to IFX or ADL [31,60,61]. In all three studies, the immunomodulators were associated with reduction and elimination of ADA titres, increases in anti-TNF trough levels, and restored clinical responses. Although only one study reported the differential effects of MTX compared to thiopurines, it found no difference inefficacy between agents on these outcomes [31]. Furthermore, the addition of an immunomodulator was more effective than dose intensification of anti-TNFs alone [61].

### 3.2. Efficacy of Methotrexate Compared to Thiopurines at Augmenting Anti-TNF Pharmacokinetics

In terms of transitioning to the preferential use of MTX for concomitant immunomodulation, the first question that must be answered is whether it is as effective as thiopurines at maintaining anti-TNF trough levels and preventing ADA formation. There have been only two prospective observational studies that have directly compared the efficacies of MTX and thiopurines in combination with anti-TNFs [19,28]. There was no difference in the anti-TNF drug levels between the groups in either study, and MTX was found to be as effective at reducing ADA formation as AZA. The first study was a prospective cohort study of 369 patients with IBD and on maintenance IFX, vedolizumab, or ustekinumab. It investigated the differences in pharmacokinetics between biologic monotherapy and combination therapy with either MTX or thiopurines at varying doses. MTX was given orally, with the majority (65.4%) in low doses of 12.5 mg/week. IFX drug levels were found to be significantly lower in those who received IFX monotherapy (3.8 µg/mL) compared to those on concomitant MTX (17.1 µg/mL, *p* = 0.0001) and thiopurines (14.5 µg/mL, *p* = 0.01), with a trend towards higher levels in the MTX group compared to the thiopurines (*p* = 0.07) [19]. The rates of ADA formation were higher in those on IFX monotherapy compared to combination therapy (OR 8.6; 95% CI 2.59–29.16); however, this was not stratified by type of immunomodulator. The second study followed a cohort of 174 patients with CD across three centres, all of whom were treated with IFX in an episodic on-demand schedule. In total, 37.3% received AZA, 28.7% received MTX, and 34% received no concomitant immunomodulator [28]. MTX was given only to those who had previous thiopurine intolerance. It was administered parenterally at 15 mg/week after a 12-week induction of 25 mg/week. Those researchers found that MTX was as effective at reducing ADA formation as AZA, and both significantly reduced the risk of ADA formation compared to IFX monotherapy. They also showed that there was no significant difference in the rate of ADAs in those who started their concomitant immunomodulators > 3 months prior to commencing IFX compared to those who started the immunomodulators at the time of IFX induction. There was no difference in the median IFX level in those receiving AZA or MTX; this was measured 4 weeks after each infusion (6.15 µg/mL vs. 5.65 µg/mL, respectively; *p* = 0.27) [28]. 

## 4. Optimal Methotrexate Dosing for Concomitant Immunomodulation

The optimal dose and route of administration of MTX to optimise anti-TNF levels and prevent ADA formation is yet to be determined. When used as a monotherapy for IBD, MTX has traditionally been given parenterally and in high doses of 15–25 mg weekly [56]. However, high-dose subcutaneous administration may be unnecessary when MTX is used for the purpose of augmenting the pharmacokinetics of anti-TNF agents (Table 1 and Table 2).

Oral MTX has been shown to be as effective as parenteral administration in improving clinical outcomes when used in combination with anti-TNFs [30,33], although there is a paucity of data available for direct comparison. A retrospective review of over 200 patients demonstrated no differences in clinical outcomes such as IBD-related hospitalisations or surgery, change in biologic therapy, and steroid initiation between concomitant oral and parenteral MTX [33]. MTX is absorbed in the proximal jejunum to a varying extent between individuals, resulting in a bioavailability ranging from 30 to 90% [62]. This was demonstrated in patients with a range of rheumatological and dermatological conditions and appears to be independent of gastrointestinal disease involvement. Indeed, two small studies of patients with quiescent CD found oral MTX to have a bioavailability of 73–86% compared to subcutaneous administration [63,64]. Evidence around potential reduced absorption in those with proximal small bowel disease is lacking [65].

The saturable, dose-dependent mechanism of MTX absorption means the bioavailability of the oral formulation is higher at lower doses of up to 15 mg [65]. This explains why MTX, at low doses of 10–12.5 mg/week, is sufficient to reduce ADA formation and increase anti-TNF levels in both rheumatology and IBD [19,29,34,35,37], as exemplified in the CONCERTO trial [35]. This large randomised double-blind parallel-armed study investigated the effects of oral MTX at 2.5 mg, 5 mg, 10 mg, and 20 mg/week doses in combination with ADL in almost 400 patients with rheumatoid arthritis. There were lower rates of ADAs with increasing doses of MTX of up to 10 mg, with corresponding increases in the mean ADL levels, of up to 6.5 µg/mL, for those on 10 mg/week compared to 4.4 µg/mL in the 2.5 mg/week group. This dose-dependent effect was limited to a ceiling of 10 mg/week, with no difference in ADL levels compared to the 20 mg/week group. Improvement in clinical disease activity also plateaued at an MTX dose of 10 mg/week. These results have been reiterated in IBD cohorts with MTX doses of 10–12.5 mg/week [19,29,33]. A prospective cohort study of patients with IBD on IFX found that concomitant oral MTX improved trough levels to 17.1 µg/mL compared to 3.8 µg/mL in those on IFX monotherapy (*p* = 0.001). The improvement in IFX levels with MTX was numerically higher than that with thiopurines (14.5 µg/mL), although this did not reach significance (*p* = 0.07). The majority of the patients in the MTX group received doses of 12.5 mg/week [19]. Similarly, a cross-sectional study of over 200 paediatric patients found that concomitant low-dose oral MTX increased IFX trough levels to 15.59 µg/mL compared to 12.35 µg/mL in those who received IFX monotherapy (*p* = 0.01) [29].

### Role of Therapeutic Drug Monitoring in Guiding Methotrexate Dosing

Although TDM and metabolite monitoring are well-established for anti-TNFs and thiopurines, there is no such role to guide MTX administration. MTX is a prodrug that only inhibits purine synthesis once it has had a number of glutamic acid residues added to it to form MTX polyglutamates [66]. Long-chain MTX-polyglutamates (MTX-PGs) are not effluxed efficiently from cells and therefore are a measure of intra-cellular MTX concentration [67]. A systematic review of the use of MTX-PG monitoring in inflammatory arthopathies has demonstrated that there may be a role for TDM in targeting disease activity but that it was not useful in predicting MTX toxicity or AEs [68]. A small cross-sectional study in a paediatric Crohn’s cohort found a trend towards increased short-chain MTX-PGs in those who were in remission compared to those with active disease [67]. Conversely, a similar retrospective study in adult IBD patients found that increased long-chain MTX-PG concentrations were associated with worse clinical disease activity and a higher rate of AEs [69]. Given this paucity of evidence, there is no established role for TDM of MTX via polyglutamate testing. 

## 5. Safety Profile of Methotrexate as a Concomitant Immunomodulator with Anti-TNF Agents

The side effect profile of thiopurines has been well-described, with a range of mild-to-moderate AEs reported [26,70]; however, it is the more serious AEs, including infections, NMSC, and lymphoma, associated with their prolonged use that cause concern. These risks are increased when thiopurines are used in combination with anti-TNF agents [27,71,72,73,74,75].

MTX has a similar mild-to-moderate side effect profile to that of thiopurines. In fact, a retrospective cohort study of almost 800 patients with IBD found that those on MTX were more likely to discontinue treatment due to nausea, fatigue, and hepatotoxicity than those on thiopurines [70]. Meanwhile, patients who took thiopurines had higher rates of pancreatitis and lower leukocyte and neutrophil counts at 1 year. The patients on MTX were older and had higher rates of prior immunomodulator intolerance compared to those on thiopurines. Oral MTX was better tolerated than subcutaneous administration, with significantly less fatigue (3% vs. 10%, respectively; *p* = 0.04) and a trend towards lower discontinuation rates (32% vs. 45%, respectively; *p* = 0.07). The researchers of that study also found that lower doses (<20 mg oral or <15 mg subcutaneous) were better tolerated, with numerically lower discontinuation rates (24% vs. 40%, respectively; *p* = 0.19) compared to higher doses (≥20 mg oral or ≥15 mg subcutaneous). Of note is that these lower doses are still higher than is required for concomitant immunomodulation with anti-TNF therapy. Supplementation with folic acid reduces the incidence of gastrointestinal side effects and hepatotoxicity, improves tolerability, and helps prevent cytopenias [76,77].

More serious but less common AEs of MTX include interstitial lung disease and pleural or pericardial serositis [78,79]. MTX is not, however, associated with lymphoproliferative disorders when used in monotherapy or in combination with anti-TNF agents [80]. Combination immunosuppressive therapy has raised concern around increasing risk of infective complications. Indeed, a population-based French study of over 190,000 IBD patients showed that concomitant thiopurine and anti-TNF therapy increased the risk of serious and opportunistic infections compared to anti-TNF monotherapy [72]. This same risk is not apparent with concomitant MTX. A large retrospective registry study of almost 8000 patients with rheumatoid arthritis reviewed the risk of infections in patients on combination MTX (mean dose of 13.2 mg/week) and anti-TNF therapy compared to monotherapy with either agent [81]. These data followed patients for 15,047 patient-years. Surprisingly, there were no increased rates of infection in those on concomitant MTX and anti-TNF compared to those on anti-TNF monotherapy (37.1/100 person-years, 95% CI [34.9–39.3] vs. 41.8/100 person-years, 95% CI [37.0–43.3], respectively) [81]. Whilst the risk may differ between rheumatoid arthritis and IBD, these data suggest that MTX may have a lower rate of infective complications than thiopurines.

## 6. Recommendations for the Use of Low-Dose Oral Methotrexate in Combination with Anti-TNF Agents

Given the potential safety benefits and demonstrated pharmacokinetic efficacy, we suggest clinicians consider using low-dose oral MTX as an alternative to thiopurines for concomitant immunomodulation with anti-TNF therapy for IBD. Low-dose oral MTX is particularly suitable when the primary aim of the concomitant immunomodulation is to reduce immunogenicity and increase anti-TNF drug levels rather than as a second therapeutic agent to treat active disease. Other clinical scenarios where low-dose MTX should be considered for concomitant immunomodulation include:

- EBV-naïve patients, especially males (due to the risk of lymphoproliferative disorders);
- Young males (due to the rare but devastating risk of hepatosplenic T-cell lymphoma);
- Thiopurine-intolerant patients;
- Homozygous thiopurine methyltransferase (TMPT)- or Nudix hydrolase-15 (NUDT15)-deficient patients.

Whilst MTX is contraindicated in pregnancy and should be discontinued at least 3 months prior to conception, it has been shown to be safe in males who are planning on fathering a child [82]. MTX should be avoided in those with chronic liver disease, and dose reductions may be required for those with renal impairment. 

## 7. Conclusions

The available evidence suggests that MTX has comparable efficacy to thiopurines in augmenting the pharmacokinetics of anti-TNF agents. It has also been demonstrated to eliminate ADAs, increase trough levels, and recapture clinical responses in those with loss of response and on anti-TNF monotherapy. There are, however, a lack of head-to-head data comparing these two agents as concomitant immunomodulators. Given the heterogeneity of the dosing regimens that have been studied to date, further investigation with more stringent subgroup analyses and consistent MTX doses will help clarify these findings. Overall, low-dose oral MTX (i.e., 10–12.5 mg weekly) is better tolerated and appears to be as effective as higher-dose parenteral administration in improving anti-TNF pharmacokinetics. Furthermore, given a potentially more favourable serious AE profile compared to thiopurines, low-dose oral MTX may be considered as an alternative first-line option for concomitant immunomodulation alongside anti-TNF therapy. 

## Figures and Tables

**Table 1:** Summary of original research reporting efficacy of low-dose MTX as a concomitant immunomodulator with anti-TNF therapy.

Study	Design	Anti-TNFs	MTX Dosing, mg/Week	Characteristics	Drug Level, µg/mL	ADA Formation	Clinical Outcomes
*Gastroenterology Studies*							
**Colman (2015) [30]**	Retrospective review	IFX ADL CZP	75% used PO MTX 25% used parenteral MTX 71% used LD-MTX (≤12.5 mg) 29% used HD-MTX (15–25 mg)	73 adult patients with IBD - 74% with CD - Active disease All on anti-TNF therapy in combination with MTX - 49% on ADL - 40% on IFX - 11% on CZP Followed for 42 months Secondary outcomes: - Endoscopic inflammation - Steroid use - Therapy escalation - Addition or escalation of concomitant therapy - Surgery	-	-	No difference in relapse rate between methods of MTX administration - 37% PO vs. 27% parenteral; *p* = 0.56 HD-MTX more likely to maintain remission than LD-MTX; log-rank test *p* < 0.01 No difference in secondary outcomes indicating worsening disease between MTX doses (OR 1.14; 95% CI 0.61–2.13; *p* = 0.67)
**Ungar (2017) [31]**	Retrospective multi-centre (3) review	ADL	Mix of PO and SC MTX (% not stated) SC dose: 15–25 mg PO dose: 10–15 mg	23 adult patients with IBD - 91% with CD All developed ADAs with LOR in ADL monotherapy; immunomodulator was added as salvage combination therapy - 14 on thiopurines - 9 on MTX	-	48% of patients had elimination of ADAs - No difference in type of immunomodulator; *p* = 0.5	Patients who had reversal of ADA achieved clinical responses and normalisation of inflammatory markers
**Chi (2018) [29]**	Cross-sectional analysis	IFX	“Primarily low dose oral MTX, mean dose 11.6 mg ± 5.1 mg/week”	223 paediatric and young adult patients with IBD - 83.9% with CD All on IFX - 62.3% as monotherapy - 37.7% as combination therapy Of the combination therapy: - 84.5% used MTX - 15.5% used 6-MP	Higher TLs in combination therapy **(15.59 ± 1.20)** vs. monotherapy **(12.35 ± 0.93); *p* = 0.01** Monotherapy **(27.3%)** more likely to have **subtherapeutic TLs < 3.5** than combination therapy **(8.3%): OR 0.13; 95% CI 0.04–0.39; *p* < 0.01** No difference in mean TL between the MTX (15.2) and MP (17.9) groups; *p* = 0.41	Combination therapy (9.5%) was less likely to result in ADAs than monotherapy (20%) (OR 0.3; 95% CI 0.1–0.7; *p* < 0.01) Trend towards higher rates of ADAs in 6-MP (23.08%) vs. MTX (7.04%) use; *p* = 0.07	No difference in clinical or biochemical disease activity between IFX monotherapy and combination therapy
**Vasudevan (2019) [32]**	Retrospective multi-centre (2) observational study	IFX ADL	PO MTX - 29% used LD-MTX (≤12.5 mg) - 71% used HD-MTX (≥15 mg)	269 adult patients with CD All on anti-TNF therapy and with ≥3 months of combination immunomodulator therapy - 58% on IFX - 42% on ADL - 71% used thiopurines - 29% used MTX	No difference in IFX TLs between thiopurines **(5.3)** and MTX **(5.4); *p* = 0.63** Higher ADL TLs with thiopurines **(7.2)** vs. MTX **(4.3)** combination therapy; ***p* = 0.03** The thiopurine combination achieved higher rates of **therapeutic ADL levels (73%)** vs. MTX **(18%); *p* < 0.01**	-	Higher rates of endoscopic remission in the ADL group with thiopurine combinations (49%) vs. MTX (6%); *p* = 0.004 No differences in remission rate between immunomodulators when used in combination with IFX - 65% on thiopurines vs. 54% on MTX; *p* = 0.09 No differences in rate of endoscopic remission between low- and high-dose MTX
**Borren (2019) [33]**	Retrospective review	IFX ADL CZP GOL	PO and SC MTX, 7.5–25 mg 28% used LD-MTX (≤12.5 mg) - 96.8% used PO 72% used HD-MTX (>12.5 mg) - 39% used PO	222 adult patients with IBD - 73.4% with CD All on anti-TNF therapy with varying doses of MTX IFX - 38.1% LD-MTX users - 37.7% HD-MTX users ADL - 44.4% LD-MTX users - 40.9% HD-MTX users CZP - 9.5% LD-MTX users - 7.0% HD-MTX users GOL - 7.9% LD-MTX users - 4.4% HD-MTX users	-	-	No difference in primary composite outcome (IBD-related hospitalisation or surgery, biological change, or steroid initiation) between the LD-MTX (37%) and HD-MTX (47%) groups; *p* = 0.15 Multi-variable analysis showed no difference in individual outcomes for either group
**Yarur (2022)** **COMBO-IBD [19]**	Prospective cohort study	IFX	PO MTX - 65.4% used LD-MTX (12.5 mg) - 34.6% used HD-MTX (25 mg)	113 adult patients with IBD - 73% with CD All on IFX - 23% on IFX monotherapy - 23% on MTX in combination - 54% on thiopurines in combination	Higher TLs in the combination MTX group **(17.1 [IQR 9.7–23.7])** and thiopurine group **(14.5 [IQR 4.5–18.8])** vs. monotherapy **(3.8 [IQR 1.8–9.2]); *p* = 0.0001** - Only those on thiopurines combined with 6-TGNs > 145 had higher TLs than in monotherapy Trend towards higher TL in MTX combination therapy than with thiopurines ***p* = 0.07**	Higher rates of ADAs in monotherapy than in combination therapy (OR8.6; 95% CI 2.58–29.16) * Not stratified by type of combination therapy	Higher rates of steroid-free deep remission in combination therapy (71.3) vs. monotherapy (46.2); *p* = 0.02 - Use of MTX and thiopurines with 6-TGNs > 145 as combination therapy were both associated with remission
*Rheumatology Studies*							
**Maini (1998) [34]**	Multi-centre randomised, double-blind placebo-controlled trial	IFX	PO MTX, 7.5 mg	101 adult patients with active RA Randomised into seven groups - IFX 1 mg/kg ± MTX - IFX 3 mg/kg ± MTX - IFX 10 mg/kg ± MTX - Placebo infusion + MTX Followed for 26 weeks	Combination with MTX showed **consistently higher drug levels** 6 weeks after the last infusion in those receiving IFX at 3 mg/kg and at 10 mg/kg IFX 1mg/kg monotherapy resulted in **undetectable TLs** from week 4 vs. stable, detectable TLs in those receiving combination MTX	Rate of ADA formation was inversely proportional to IFX dose MTX combination reduced ADA formation - 53% of IFX 1 mg/kg vs. 15% with MTX - 21% of IFX 3 mg/kg vs. 7% with MTX - 7% of IFX 10 mg/kg vs. 0% with MTX	IFX (1 mg/kg) monotherapy was no better than placebo - MTX combination achieved clinical responses in >60% of the group for a median of 16.5 weeks; *p* = 0.006 vs. IFX (1 mg/kg) monotherapy
**Burmester (2013)** **CONCERTO [35]**	Randomised, double blind parallel-armed study	ADL	PO MTX: 2.5 mg 5 mg 10 mg 20 mg	395 adult patients with RA All on ADL Randomised 1:1:1:1 to combination therapy with different MTX doses Followed for 26 weeks	Higher TLs with increasing MTX doses of up to 10 mg/week - Mean TLs of **4.4 (±5.2), 5.7 (±4.9), 6.5 (4.4), 6.9 (3.4)** for MTX at 2.5 mg, 5 mg, 10 mg, and 20 mg, respectively	Lower rates of ADAs with increasing MTX doses of up to 10 mg/week - 21%, 13%, 6%, and 6% for MTX 2.5 mg, 5 mg, 10 mg, and 20 mg, respectively	Reduced disease activity with increasing MTX doses - Higher proportion of patients meeting the primary endpoint with increasing MTX doses; *p* = 0.005
**Ducourau (2020) [36]**	Multi-centre randomised trial	ADL	SC MTX, 10 mg	107 adult patients with axial spondylarthritis All on ADL Randomised 1:1 to ADL monotherapy vs. combination with MTX - 51.4% monotherapy - 48.6% combination Followed for 26 weeks	MTX combination therapy was associated with **higher TLs** at all time points; *p* < 0.05 Those with ADAs had lower median TLs **(1.43 [0.00–11.47])** compared to those without ADAs **(8.66 [0.05–18.31])** at week 26; ***p* < 0.05**	Lower rates of ADAs in MTX combination therapy (25%) vs. ADL monotherapy (47.3%); *p* = 0.03 - MTX combination therapy reduced risk of ADA formation; RR 0.53 (95% CI 0.31–0.91)	Similar rates of clinically inactive disease by week 26 for both groups (40% ADL monotherapy vs. 37%; *p* = 0.9)
*Dermatology Studies*							
**van der Kraaij (2022) [37]**	Randomised control trial	ADL	PO MTX, 10 mg	61 adult patients with psoriasis All on ADL Randomised 1:1 to ADL monotherapy vs. combination with MTX - 49% monotherapy - 51% combination	No difference in median TL between groups; ***p* = 0.26** **- 5.9 [3.5–8.8]** for monotherapy **- 6.8 [5.5–9.2]** for combination More monotherapy patients failed to reach **therapeutic TLs > 3.2** at week 5 **(6.5%)** vs. with combination therapy **(30%); *p* = 0.02** - This was not significant at week 49; 12.9 vs. 23.3%; *p* = 0.32	Higher rates of ADA formation in monotherapy group (60%) vs. combination therapy group (22.6%); *p* < 0.01 ADAs appeared earlier in monotherapy group vs. combination therapy group - Week 5: 33.3 vs. 3.2%; *p* < 0.01 - Week 49: 46.7 vs. 38.7%; *p* = 0.31	Combination therapy had faster clinical improvement, with 83.9% achieving treatment goals in week 13 vs. 56.7% of monotherapy patients; *p* = 0.03

Abbreviations—Anti-TNF (anti-tumour necrosis factor), IFX (infliximab), ADL (adalimumab), CZP (certolizumab pegol), GOL (golimumab), MTX (methotrexate), PO (oral), SC (subcutaneous), LD (low-dose), HD (high-dose), IBD (inflammatory bowel disease), CD (Crohn’s disease), RA (rheumatoid arthritis), LOR (loss of response), TL (trough level), OR (odds ratio), CI (confidence interval), IQR (inter-quartile range), 6-MP (mercaptopurine), 6-TGNs (6-thioguanine nucleotide). * is to indicate a point of interest/qualifying remark relating to the above comment.

**Table 2:** Summary of original research reporting efficacy of high-dose MTX as a concomitant immunomodulator with anti-TNF therapy for IBD.

Study	Design	Anti-TNFs	MTX Dosing, mg/Week	Characteristics	Drug Level, µg/mL	ADA Formation	Clinical Outcomes
**Vermeire (2007) [28]**	Multi-centre (3) prospective cohort study	IFX	SC MTX at 15 mg (12-week induction with 25 mg)	174 adult patients with IBD All commenced IFX (episodic, on-demand regime) - 34% with IFX monotherapy - 37.3% with AZA combination - 28.7% with MTX combination	Higher median IFX levels in combination therapy **(6.45)** vs. monotherapy **(2.42); *p* = 0.065** - No difference between MTX **(5.65)** and AZA **(6.15); *p* = 0.27**	Lower rates of ADA formation in combination therapy (46%) vs. monotherapy (73%); *p* < 0.001 MTX (44%, *p* = 0.002) and AZA (48%, *p* = 0.004) had equal efficacies against ADA formation vs. monotherapy No difference in rate of ADA when immunomodulator started at time of IFX vs. preceding 3 months	-
**Feagan (2014)** **COMMIT [58]**	Double-blind, placebo-controlled, randomised trial	IFX	SC MTX at 25 mg (escalated from 10 mg to 25 mg over 5 weeks)	126 adult patients with CD All commenced IFX Randomised 1:1 - IFX monotherapy (placebo) - MTX combination	Trend towards higher TLs in MTX group **(6.35)** vs. monotherapy **(3.75); *p* = 0.08**	Lower rates of ADA formation in MTX group (4%) vs. monotherapy (20%); *p* = 0.01	No difference in rate of treatment failure at week 50 between MTX (30.6%) and monotherapy (29.8%) groups, (HR, 1.16; 95% CI 0.62–2.17)
**Kappelman (2023) [59]**	Multi-centre (35), placebo-controlled, randomised trial	IFX ADL	Weight-based PO MTX - 15 mg if >40 kg - 12.5 mg if 30–40 kg - 10 mg if 20–30 kg	297 paediatric (age < 21) patients with CD All commenced either IFX or ADL - 71% IFX - 29% ADL Randomised 1:1 and stratified by anti-TNF - IFX monotherapy (placebo) - MTX combination Followed for 1–3 years	-	No difference in ADA formation with MTX (34%) vs. placebo (47%) group for IFX (RR 0.72, 95% CI 0.49–1.07) No difference for ADL; 15% with MTX vs. 21% with placebo (RR, 0.71 95% CI 0.24–2.07) Those on ADL with ADAs were more likely to have treatment failure (64% vs. 36%, *p* = 0.03) * Serum available for only 70% of patients	MTX use in those on ADL reduced the risk of treatment failure vs. placebo; HR 0.40 (95% CI 0.19–0.81, *p* = 0.01) No significant differences between groups for those on IFX

Abbreviations—Anti-TNF (anti-tumour necrosis factor), IFX (infliximab), ADL (adalimumab), MTX (methotrexate), PO (oral), SC (subcutaneous), IBD (inflammatory bowel disease), CD (Crohn’s disease), TL (trough level), HR (hazard ratio), RR (relative risk), CI (confidence interval). * is used to indicate a point of interest/qualifying remark relating to this section.
